# Combinations of Independent Dominant Loci Conferring Clubroot Resistance in All Four Turnip Accessions (*Brassica rapa*) From the European Clubroot Differential Set

**DOI:** 10.3389/fpls.2018.01628

**Published:** 2018-11-13

**Authors:** Arvind H. Hirani, Feng Gao, Jun Liu, Guohua Fu, Chunren Wu, Peter B. E. McVetty, Robert W. Duncan, Genyi Li

**Affiliations:** ^1^Department of Plant Science, University of Manitoba, Winnipeg, MB, Canada; ^2^Monsanto Canada Inc., Winnipeg MB, Canada

**Keywords:** European clubroot differential set, European turnips, clubroot resistance, resistance gene mapping, *Brassica rapa*

## Abstract

Clubroot disease is devastating to *Brassica* crop production when susceptible cultivars are planted in infected fields. European turnips are the most resistant sources and their resistance genes have been introduced into other crops such oilseed rape (*Brassica napus* L.), Chinese cabbage and other *Brassica* vegetables. The European clubroot differential (ECD) set contains four turnip accessions (ECD1–4). These ECD turnips exhibited high levels of resistance to clubroot when they were tested under controlled environmental conditions with Canadian field isolates. Gene mapping of the clubroot resistance genes in ECD1–4 were performed and three independent dominant resistance loci were identified. Two resistance loci were mapped on chromosome A03 and the third on chromosome A08. Each ECD turnip accession contained two of these three resistance loci. Some resistance loci were homozygous in ECD accessions while others showed heterozygosity based on the segregation of clubroot resistance in 20 BC_1_ families derived from ECD1 to 4. Molecular markers were developed linked to each clubroot resistance loci for the resistance gene introgression in different germplasm.

## Introduction

Clubroot caused by an obligate biotrophic parasite, *Plasmodiophora brassicae* (Woronin), is recently one of the most economically important diseases of canola/rapeseed and other *Brassica* vegetable crops in the world. This disease was recorded in Japan in 1890s (Ikegami et al., 1981), and now it is known to occur in *Brassica* crops in more than 60 countries. Annually, clubroot causes significant damage to the *Brassica* vegetables and oilseed crops and 10–15% yield losses were reported due to clubroot disease in each *Brassica* crop around the world (Diederichsen et al., 2009). Clubroot disease was a major problem in *Brassica* vegetable crops for several decades, but recently this disease is spreading in *Brassica* oilseed crops in Europe, China, India, Nepal, and Australia (Wallenhammar, 1996; Manzanares-Dauleux et al., 2000; Dixon, 2009). Since clubroot disease on canola was first identified in the Canadian prairies in 2003 (Gossen et al., 2013), clubroot infected canola fields have quickly increased due to the lack of effective management practices. In the central region of Alberta, a clubroot survey revealed that there are over a thousand clubroot infested canola fields. Also clubroot disease has been identified in the canola fields of Saskatchewan and Manitoba, suggesting that this disease is spreading across the Canadian prairies. In clubroot infected fields, 60–90% yield losses were reported when susceptible canola/rapeseed cultivars were planted in 1998–2000 (Dixon, 2009). This suggests that cultivation of clubroot resistant canola/rapeseed cultivars is the most effective and sustainable management method for clubroot disease.

*Plasmodiophora brassicae* is a protist and a member of the Cercozoa phylum belonging to the subgroup of Rhizaria (Siemens et al., 2009; Niwa et al., 2011). The pathogen completes its life cycle in two phases: the first phase of life cycle takes place in the root hairs and the second phase occurs in the stele and cortex of hypocotyls and roots. In the second phase of life cycle, abnormal proliferation of tissues in the infected roots produces galls (clubs) caused by the secondary plasmodia (Ikegami et al., 1981). The galls on roots prevent water and nutrient uptake that retards normal growth and development of plants, resulting in significantly reduced yield and quality. Both phases of a life cycle can occur in resistance and susceptible plants, while development of plasmodia is inhibited or delayed in resistant plants (Kobelt et al., 2000; Hatakeyama et al., 2013). The clubroot pathogen produces oospores that are viable for several years in soil and spread through machinery and other means of soil movement such as soil erosion by wind and water (Kuginuki et al., 1999). The pathogen produces two types of spores including zoospores and long-lived resting spores, oospores. The existence of long-lived viable spores in soil increases the difficulty of managing the disease through crop rotation. Thus, it is critical to identify resistance genes and integrate these genes into current canola/rapeseeds cultivars.

Sources of currently known clubroot resistance (R) genes are from European turnip (*Brassica rapa* L. spp. *rapifera*) that carry strong resistance to clubroot (Matsumoto et al., 1998). The resistance to clubroot is suggested to be controlled by single to several R genes (Yoshikawa, 1983; Manzanares-Dauleux et al., 2000). In Japan, clubroot resistant Chinese cabbage cultivars have been developed by crossing with different accessions of European turnip (Matsumoto et al., 2005). Among clubroot resistant European turnip, several cultivars such as Gelria R, Siloga, Debra, and Milan White are the primary sources for breeding clubroot resistant Chinese cabbage cultivars in Japan and Korea.

Mapping qualitative resistance and quantitative trait loci (QTL) revealed several dominant loci in *B. rapa*, more than 22 QTL in *Brassica oleracea* and over 16 QTL in *Brassica napus* for clubroot resistance to different isolates of *P. brassicae* (Diederichsen et al., 2009; Piao et al., 2009; Hatakeyama et al., 2013). Two loci, *Crr1* and *Crr2* were identified through QTL analysis for clubroot resistance in the F_2_ population of a cross between G004 and Hakusai Chukanbohon Nou 7 in *B. rapa* (Suwabe et al., 2003). Subsequently, fine mapping of *Crr1a* suggested that the *Crr1a* locus is involved in clubroot resistance for the race Ano-01 of *P. brassicae* in *B. rapa* (Hatakeyama et al., 2013). Another locus, *CRa* was identified on linkage group A03 in a doubled haploid line T136-8 of Chinese cabbage, and this locus displayed strong dominant resistance to clubroot caused by isolate M85 of *P. brassicae* (Matsumoto et al., 1998). Similarly, a QTL mapping study identified two loci, *CRk* and *CRc* in two F_2_ populations derived from crosses of K10 × Q5 and C9 × R6 in Chinese cabbage (Sakamoto et al., 2008). The locus *CRk* was resistant to clubroot isolates, M85 and K08 whereas, clubroot resistance locus *CRc* was resistant to clubroot isolate, K08. *Crr3* was mapped through QTL analysis using the F_3_ population of a cross between a CR-turnip inbred line, N-WMR-3 and a Chinese cabbage doubled haploid line, A9709 (Saito et al., 2006). This locus was resistant to clubroot isolate, Ano-01. In addition, two clubroot resistance loci, *Crr4* (Suwabe et al., 2006) and *CRb* (Piao et al., 2004) were mapped in Chinese cabbage. In *B. napus*, a QTL mapping study reported a Mendelian gene resistance for isolate, Pb137–522 and minor QTL resistance for isolate, K92-16 in the DH population of a cross between a resistant cultivar Darmor-bzh and a susceptible cultivar Yudal (Manzanares-Dauleux et al., 2000). All these loci identified in different studies showed race-specific resistance to clubroot (Piao et al., 2009). Recent molecular characterization of the *CRa* locus on A03 revealed that TIR-NBS-LRR genes are responsible for clubroot resistance to isolate M85 of *P. brassicae* (Ueno et al., 2012). Similarly, another locus, *Crr1* on chromosome A08 also has an R gene coding for a *TIR-NBS-LRR* protein (Hatakeyama et al., 2013).

Long term cultivation of clubroot disease resistant Chinese cabbage cultivars and hybrids has resulted in breakdown of the effectiveness of resistance genes in Japan, Korea, and China due to the evolution of *P. brassicae* pathogenicity. Genetics of clubroot disease resistance in original sources need to be investigated for the identification of novel clubroot resistance loci. In this study, mapping of clubroot resistance genes in turnip accessions of the European clubroot differential (ECD) set was performed and molecular markers linked to clubroot resistance genes in these ECD turnips were developed for marker-assisted selection in canola and other *Brassica* species. Among the clubroot resistant ECD set, allelic variation was investigated to develop allele-specific molecular markers for marker-assisted selection in canola/rapeseed breeding.

## Materials and Methods

### Plant Materials

The ECD set was obtained from Warwick HRI’s Vegetable Genebank, United Kingdom. The ECD set includes five accessions of *B. rapa* (ECD1–5), five accessions of *B. napus* (ECD6–10) and five accessions of *B. oleracea* (ECD11–15). These 15 accessions were evaluated to identify clubroot disease resistant materials against Canadian field isolates. After controlled environment evaluation, clubroot resistance in four turnips, ECD1, ECD2, ECD3, and ECD4 were identified and used as resistant male parents to cross with a susceptible female parent of *B. rapa* accession, yellow sarson (YS). The clubroot resistant turnips were winter types, with brown seed coat color and self-incompatible. In contrast, the yellow sarson was susceptible to clubroot, with yellow seed, spring growth habit and self-compatibility characteristics. Four crosses YS × ECD1, YS × ECD2, YS × ECD3, and YS × ECD4 were made in the greenhouse at the University of Manitoba after 6 weeks of vernalization for all four ECDs. Subsequently, five F_1_ individual plants from each cross were used to make backcrosses to the susceptible parent (YS) to produce a total 20 BC_1_ families. Since clubroot resistant turnips were self-incompatible and open pollinated accessions therefore genetic purity was unknown. All the 20 BC_1_ families were produced from individual F_1_ plants separately and further phenotypic evaluation was performed on the family basis in each of these four BC_1_ populations.

### Clubroot Disease Evaluation and Statistical Analysis

Clubroot disease evaluation was performed in greenhouses by Monsanto Canada at the University of Guelph, Guelph, ON, Canada using Canadian field isolates. The clubroot pathogen was collected from infected canola fields in AB, Canada. One part infected soil was thoroughly mixed with three parts Metro-Mix soil (Sun Gro^®^ Horticulture, Canada) for even distribution of pathogen resting spores. Ten to 12 seeds were planted in 15 cm pots with three replicates per genotype. Resistant and susceptible parental genotypes were seeded as controls in each experiment. Six weeks after inoculation, seedlings were classified into four groups corresponding to four levels of resistance based on the distribution of galls on roots. A score of zero was given for plants without galls on both primary and secondary roots, one for plants with a few small galls on the secondary roots, two for plants with a few small galls on both primary and secondary roots and three for plants with several big galls on both primary and secondary roots. Groups 0 and 1 were considered clubroot resistant reactions, and groups 2 and 3 as clubroot susceptible reactions for phenotype evaluation of BC1 families. Segregation of clubroot disease resistant and susceptible plants in each family of BC_1_ populations was analyzed using the Chi-square (χχ^2^) test for goodness-of-fit.

### DNA Extraction and PCR for Polymorphism Detection

Plant genomic DNA was extracted using a modified CTAB method from young leaves of parental genotypes and segregating populations (Li and Quiros, 2001). Genomic DNA was used for PCR amplification and polymorphism detection. SRAP PCR amplification was performed as described by Li and Quiros (2001). Standard PCR was carried out for SSR and SCAR amplification with M13 tailed forward primers and M13 labeled primer with four different colors (Gao et al., 2014). SRAP and SSR PCR products were separated with an ABI 3130xl Genetic Analyzer (ABI, CA, United States) and five fluorescent dye set, 6-FAM (blue), VIC (green), NET (yellow), PET (red), LIZ (orange) was used for signal detection (Life Technologies, Canada). The LIZ orange color fluorescent dye was used as a size standard with the other four colors for PCR product separation.

### Analysis of the Clubroot Resistance Gene Specific Molecular Markers on Chromosome A03

Fine mapping of clubroot resistance gene in Chinese cabbage was previously carried out and molecular markers linked to the gene on chromosome A03 were developed (Gao et al., 2014). For better clarification and consistency with *Brassica* gene nomenclature, previously fine mapped gene in Chinese cabbage was designated here as *BraA.CR.a.* These molecular markers were employed to determine haplotypes of resistance genes on chromosome A03. Four SSR markers, FSASS45c, FSASS79b, FSASS72b, and FSASS45b were used to select clubroot resistant individual plants with clubroot resistance genes on chromosome A03. These SSR markers were analyzed in all BC_1_ segregating populations developed from each of ECD clubroot resistance parental genotypes. Data were analyzed by the ABI GeneScan software and visualized by Genographer software to score genotypes.

### Mapping of Other Clubroot Resistance Genes in ECDs

Further analysis focused on the BC_1_ genotypes, which did not show co-segregation with gene specific molecular markers on chromosome A03, or when the segregation ratio was not 1:1. From the selected BC_1_ genotypes, eight resistant plants and eight susceptible plants from each line were used for bulk segregant analysis using SRAP molecular markers. Bulk segregant analysis was performed using a total 150 SRAP primer pairs. SRAP PCR was performed using the standard protocol described by Li and Quiros (2001).

In bulk segregant analysis, SRAP markers associated with clubroot phenotypes were identified and their PCR products were sequenced. The sequence information of these SRAP markers was compared against the *B. rapa* genome sequence^1^. Genomic regions between extreme SRAP marker sequences were extracted from the *B. rapa* genome sequence using BEDtools with Linux commands (Quinlan and Hall, 2010). Subsequently, blastx analysis was carried out for the extracted genomic regions on the NCBI database with least Blast Expected Value of 1.10^-3^ using the blast2go software v2.5. Predicted candidate genes were identified within 2 Mb region and new SSR markers were developed to analyze the candidate clubroot resistance loci.

## Results

### Clubroot Disease Resistance for the ECD Set of *Brassica* Species

The objective of this study was to map clubroot resistance gene in all ECD set and develop molecular markers for each clubroot resistance gene for introgression in other *Brassica* species in breeding programs. The European clubroot differential set (ECD1–15) was evaluated for clubroot resistance using Canadian clubroot field isolates. Among the five *B. rapa* accessions, all four turnips, ECD1–4 were resistant while one Chinese cabbage ECD5 was susceptible (Table 1). Among five *B. napus* accessions, most plants in these four rapeseed accessions, ECD6–9 were susceptible, but most plants in *B. napus* rutabaga, ECD10 showed obvious resistance. In *B. oleracea*, two cabbage accessions, ECD11 and ECD12 were resistant and ECD13 and ECD15 segregated while most plants in ECD14 were susceptible. All data suggested that clubroot resistance in *B. rapa* turnips was stronger than that in other *B. napus* and *B. oleracea* accessions (Table 1).

**Table 1:** Evaluation of clubroot disease resistance of European clubroot differential sets (ECD1–15) with Canadian clubroot field isolates.

ECD	Clubroot ratings			
	
accessions	0	1	2	3
ECD1	35	0	0	0
ECD2	43	0	0	0
ECD3	39	0	0	0
ECD4	43	0	0	0
ECD5	0	2	2	43
ECD6	4	0	2	35
ECD7	0	2	2	29
ECD8	2	0	1	29
ECD9	9	3	3	21
ECD10	24	0	0	4
ECD11	41	0	0	0
ECD12	22	0	0	0
ECD13	12	0	6	13
ECD14	0	2	2	16
ECD15	18	2	4	9


*Clubroot disease scoring: 0, plant without galls on both primary and secondary roots; 1, plants with small galls on the secondary roots; 2, plant with small galls on both primary and secondary roots; 3, plant with large galls on both primary and secondary roots.*

### Evaluation of Clubroot Disease Resistance in BC_1_ Populations of *B. rapa*

The following analysis focused on four turnip accessions in the ECD set (ECD 1–4). Since all four turnips showed resistance to the Canadian clubroot isolates, four mapping populations were developed by crossing these four turnips with a susceptible *B. rapa* yellow sarson. All four BC_1_ segregating populations were phenotyped and classical genetic analysis of the phenotypes in these four segregating populations suggested that one and two gene models explained all ratios of the clubroot resistance segregation (Table 2). One gene segregated in nine BC_1_ lines derived from original individual plants of ECD1, ECD2, and ECD3 while two genes segregated in the other five BC_1_ lines derived from these three ECD accessions, suggesting that the ECD1, ECD2, and ECD3 accessions all contained a mixture of plants. In this mixture, some plants had one clubroot resistance locus and others had two clubroot resistance loci. In the mapping population derived from ECD4, all BC_1_ families showed a two gene segregation ratio of 3:1, suggesting that all ECD4 plants carried two clubroot resistance loci (Table 2). The classical genetic analysis revealed that there were various inheritances of clubroot disease resistance loci in all turnips of the ECD set in this study.

**Table 2:** Segregation of clubroot disease in BC_1_ families of a cross between ECD (1–4) and susceptible parent (YS) in *Brassica rapa*.

Pedigree	Phenotype		Total	χ^2^	*P*-value	Ratio^∗^	*df*
					
	Resistant	Susceptible					
YSx(YSxECD1-4)	24	22	46	0.09	0.77	1:1	1
YSx(YSxECD1-6)	39	37	76	0.05	0.82	1:1	1
YSx(YSxECD1-7)	14	15	29	0.03	0.85	1:1	1
YSx(YSxECD2-4)	22	18	40	0.40	0.53	1:1	1
YSx(YSxECD2-5)	15	21	36	1.00	0.32	1:1	1
YSx(YSxECD2-7)	40	33	73	0.67	0.41	1:1	1
YSx(YSxECD3-2)	68	65	133	0.07	0.79	1:1	1
YSx(YSxECD3-4)	26	20	46	0.78	0.38	1:1	1
YSx(YSxECD3-5)	28	22	50	0.72	0.40	1:1	1
**Total**				3.82	0.93		9
Goodness of fit χ^2^	276	253	529	1.00	0.32	1:1	1
Homogeneity χ^2^ (Total- Pooled data)				2.82	0.95		8

YSx(YSxECD1-2)	49	10	59	2.04	0.15	3:1	1
YSx(YSxECD2-2)	26	9	37	0.04	0.80	3:1	1
YSx(YSxECD3-1)	89	30	119	0.00	0.96	3:1	1
YSx(YSxECD3-3)	127	39	166	0.20	0.65	3:1	1
YSx(YSxECD3-6)	133	38	171	0.70	0.40	3:1	1
YSx(YSxECD3-7)	40	15	55	0.15	0.70	3:1	1
YSx(YSxECD4-l)	30	6	36	1.33	0.25	3:1	1
YSx(YSxECD4-2)	14	5	19	0.02	0.89	3:1	1
YSx(YSxECD4-4)	27	9	36	0.00	1.00	3:1	1
YSx(YSxECD4-5)	52	21	73	0.55	0.46	3:1	1
YSx(YSxECD4-8)	57	16	73	0.37	0.54	3:1	1
**Total**				5.55	0.94		12
Goodness of fit χ^2^	644	198	842	0.43	0.51	3:1	1	
Homogeneity χ^2^ (Total- Pooled data)				4.95	0.40		11	


*^∗^Ratios represent one and two clubroot resistance genes segregating families were combined in each BC_1_ population derived from the same ECD accession as resistance parent.*

### Association of Clubroot Resistance Genes in the BC_1_ Populations

All BC_1_ families were screened using four SSR markers (FSASS45c, FSASS79b, FSASS72b, and FSASS45b) linked to the previously mapped clubroot resistance locus, *BraA.CR.a* on chromosome A03. All four SSR markers co-segregated with the clubroot resistance in the BC_1_ families where clubroot resistant and susceptible plants segregated at a ratio of 1:1 in three BC_1_ families derived from ECD1 (Table 2). In the BC_1_ families segregating at a ratio of 3:1 for clubroot resistance, the yellow sarson susceptible alleles of all SSR markers for *BraA.CR.a* on A03 were homozygous in all susceptible individual plants. The resistant alleles from ECD1 segregated as heterozygotes in some resistant individuals, indicating that the A03 clubroot resistance locus existed in all ECD1 individual plants. Another new clubroot resistance locus segregated in the genotypes segregating at a ratio of 3:1 (Table 2).

In three ECD2 BC_1_ families segregating with a 1:1 ratio, the SSR markers specific to the *BraA.CR.a* locus on A03 did not co-segregate with clubroot resistant and susceptible phenotypes, indicating that the A03 clubroot resistance locus did not exist in some ECD2 plants. In the genotypes segregating with a 3:1 ratio, the yellow sarson alleles were homozygous in all susceptible plants while the ECD2 alleles of all SSR markers on A03 were both homozygous and heterozygous in different individual plants, indicating that the A03 resistance locus segregated in the ECD2-derived families. Therefore, in ECD2, the A03 resistance locus existed in some individual plants, but not others. Additionally, a new resistance locus existed in ECD2 according to the segregation ratios of 1:1 and 3:1 (Table 2).

In the three ECD3 BC_1_ families segregating with a 1:1 ratio of susceptible and resistant plants, all A03 SSR markers showed linkage to the A03 resistance locus, but did not co-segregate with the resistant phenotype. This indicates that a new resistance locus in these families was located on the A03 chromosome, but not the same locus, *BraA.CR.a* as mapped by Gao et al. (2014). In all three ECD3 BC_1_ families segregating with a 3:1 ratio of susceptible and resistant plants, the yellow sarson alleles were homozygous in all susceptible plants, indicating that the two new resistance loci segregated in these three BC_1_ families. Therefore, the mapping data suggested that previously mapped clubroot resistance locus, *BraA.CR.a* did not co-segregate with the resistant phenotype in ECD3. Both clubroot resistance loci in ECD3 need to be mapped in ECD3 derived BC_1_ families segregating with a 1:1 and a 3:1 ratio of clubroot resistant and susceptible phenotype.

The phenotypic data suggested that two genes segregated in all five ECD4 BC_1_ families. All yellow sarson alleles of four SSR markers for the clubroot resistance locus *BraA.CR.a* on A03 were homozygous in all susceptible plants of all five BC_1_ families. In the resistant plants, these ECD4 alleles for the A03 SSR markers were homozygous in some individual plants, but heterozygous in others. This indicated that the A03 clubroot resistance locus and another new clubroot resistance locus segregated in the ECD4 BC_1_ families (Table 4).

### Association of SRAP Markers on a Consensus Map

**FIGURE 1:**
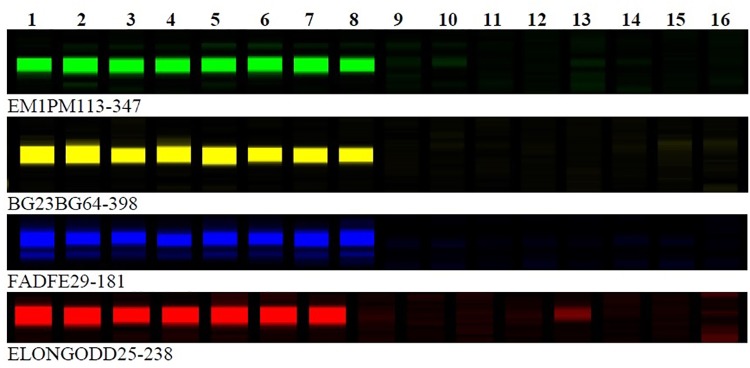
SRAP molecular marker association with clubroot resistance genes in BC_1_ populations of *Brassica rapa*. Lane 1 to 8 represent clubroot resistance individuals and lane 9–16 absence of band represent clubroot susceptible individuals in BC_1_ populations of *Brassica rapa*.

To identify the other clubroot resistance loci, the BC_1_ resistant plants that did not carry the A03 resistance alleles from ECD accessions were used in bulk segregant analyses. To map clubroot resistance loci on the previous ultra-dense genetic map in *B. rapa* (Li et al., 2011), 150 out of 243 SRAP primer pairs were selected to identify SRAP markers linked to clubroot resistance loci. Sixteen BC_1_ plants (eight clubroot resistance and eight clubroot susceptible) were screened with 150 SRAP primer pairs. Interestingly, five SRAP markers, FADFE01-388, FADFE29-181, EM1PM113-347, BG23BG64-398, and ELONGODD25-238 were found to be linked to a new clubroot resistance locus (Figure 1). These five SRAP markers were then tested on all clubroot resistance and susceptible plants which did not carry the A03 resistance locus, *BraA.CR.a*. Since the SRAP primer pairs and the susceptible parent in the ECD derived populations for mapping new clubroot resistance loci were the same as those used in the construction of the ultra-dense genetic map in *B. rapa* (Li et al., 2011), it was feasible to identify linkage groups anchoring the clubroot resistance loci based on the locations of the SRAP markers linked to clubroot resistance loci which were polymorphic on the genetic map. After comparing the SRAP markers linked to the new clubroot resistance locus with the SRAP markers on the ultra-dense genetic map, three SRAP markers were located on chromosome A08 (2 in bin R08b32 and 1 in bin R08b22) of ultra-dense genetic map, respectively (Li et al., 2011). To identify physical positions of these linked SRAP markers, these SRAP markers were sequenced and blasted onto the *B. rapa* genome sequence (see text footnote 1) to find chromosomal regions anchoring these SRAP markers. Interestingly, SRAP marker sequences showed blast hits on chromosome A08 and thus, the genomic sequence flanking these linked SRAP markers were used to develop new SSR markers and identify candidate genes.

### Development of Microsatellite Markers for the Clubroot Resistance Locus on Chromosome A08

**FIGURE 2:**
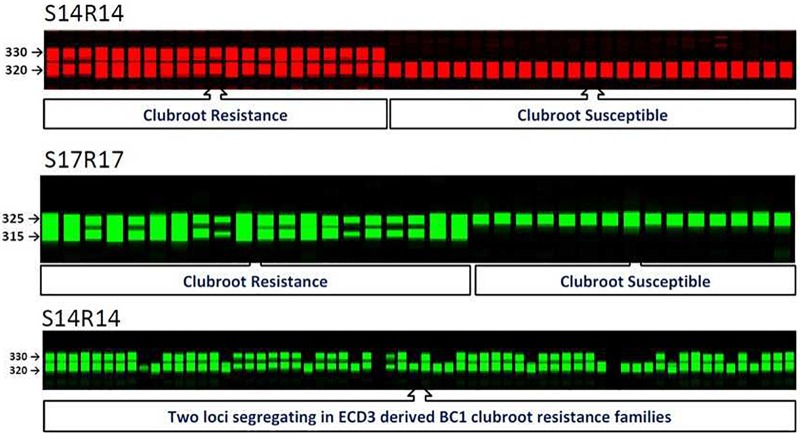
SSR molecular markers and their co-segregation with clubroot resistance locus on A08 in BC_1_ populations of *Brassica rapa*. S14R14 gel image displayed segregation of new clubroot resistance locus in clubroot resistance ECD3 families.

**FIGURE 3:**
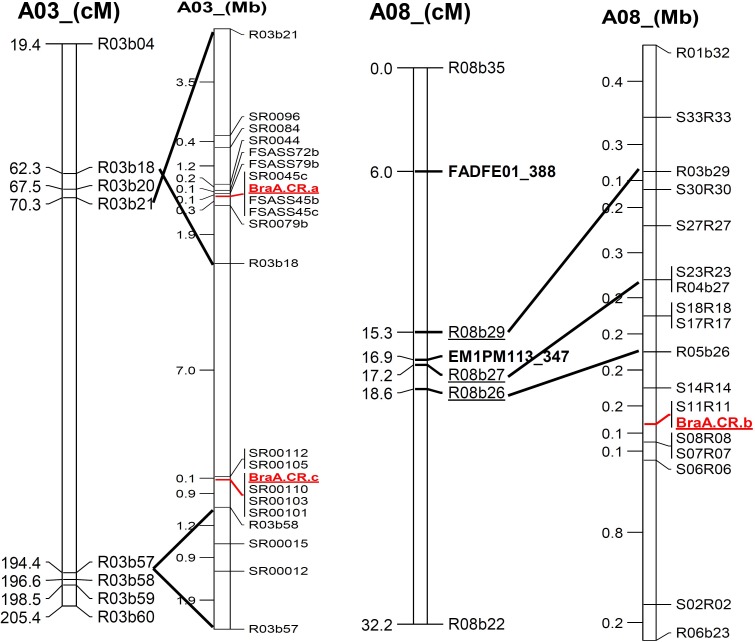
Alignment of genetic linkage map of chromosomes, A03 and A08 based on SRAP marker sequence to physical position of newly developed SSR marker along with three clubroot resistance loci mapped in *Brassica rapa*.

Genome sequence information of *B. rapa* that flanked SRAP markers linked to the candidate clubroot resistance gene was used to design new SSR markers. In total, 34 SSR markers were developed and these SSRs covered approximately 2 Mb of the *B. rapa* genome (Table 3 and Figures 2, 3; see text footnote 1). Out of 34 SSR primer pairs, 19 (57%) produced polymorphic loci between parental genotypes and were used for further analysis with clubroot disease resistance in all four BC_1_ populations. Twelve out of the 19 primer pairs (63%) were found to be linked to the clubroot resistance locus. Eight SSR markers were co-dominant and four SSR markers, dominant (Tables 3, 6). Three co-dominant SSR markers were used to test all BC_1_ populations derived from the four ECD clubroot resistance parents (ECD1–4). This new clubroot resistance locus was named as *BraA.CR.b* in *B. rapa* following the *Brassica* species gene nomenclature requirement (Østergaard and King, 2008).

**Table 3:** Microsatellite markers linked to three clubroot resistance loci on chromosome A03 and A08 in *Brassica rapa*.

SSR marker	Chromo	Physical	Nature of
	some	position (Mb)	markers
S33R33	A08	9.06	Dominant
S30R30	A08	9.46	Dominant
S27R27	A08	9.74	Dominant
S23R23	A08	9.96	Co-dominant
S18R18	A08	10.17	Co-dominant
S17R17	A08	10.23	Co-dominant
S14R14	A08	10.56	Co-dominant
S11R11	A08	10.78	Co-dominant
S08R08	A08	10.93	Co-dominant
S07R07	A08	10.94	Co-dominant
S06R06	A08	10.97	Co-dominant
FSASS45c	A03	24.30	Dominant
FSASS79b	A03	24.40	Dominant
FSASS72b	A03	24.50	Dominant
FSASS45b	A03	24.30	Co-dominant
SS15FR1	A03	15.03	Co-dominant
SS15FR3	A03	15.04	Co-dominant
SS15FR5	A03	15.06	Co-dominant


In all three ECD1-derived BC_1_ families that showed a segregation ratio of 3:1 (Table 3), all yellow sarson alleles of three SSR markers for *BraA.CR.b* on A08 showed co-segregation in all susceptible plants while the ECD1 resistant alleles were homozygous or heterozygous in the resistant plants, indicating that *BraA.CR.b* was the second resistance locus. Therefore, in the ECD1 accession, the A03 resistance locus was homozygous while the new A8 resistance locus was segregating.

Among all three ECD2-derived BC_1_ families that showed 1:1 segregation ratios, three A08 SSR markers co-segregated with the phenotypes of all progeny, suggesting that this resistance locus, *BraA.CR.b* conferred clubroot resistance in these BC_1_ families. These three SSR markers on A08 were used to test the ECD2 BC_1_ families segregating with a 3:1 ratio. The data showed that the A08 resistance locus, *BraA.CR.b* was one of two resistance loci segregating in these BC_1_ families since all yellow sarson alleles were homozygous in all susceptible progeny and the ECD2 alleles segregated with the resistant individual plants. Thus, the new A08 resistance locus was homozygous while the A03 resistance locus was heterozygous in ECD2.

Among all three ECD3-derived BC_1_ families that showed 1:1 segregation ratios, three A08 SSR markers co-segregated with the phenotypes of all progeny, suggesting that this resistance locus, *BraA.CR.b* controlled clubroot resistance in these BC_1_ families. These three SSR markers on A08 were used to test the four ECD3 BC_1_ families segregating with a 3:1 ratio (Table 2). The data showed that the A08 resistance locus, *BraA.CR.b* was one of two resistance loci segregating in these BC_1_ families since all yellow sarson alleles were homozygous in all susceptible progeny and the ECD3 alleles segregated with the resistant individual plants. Thus, the A08 resistance locus, *BraA.CR.b* was homozygous while the new resistance locus was revealed to be heterozygous that needs to be mapped in ECD3 (Figure 2).

In the ECD4-derived BC_1_ families, the two resistance loci existed in a homozygous state since all five BC_1_ families exhibited a 3:1 ratio. The previous analysis of SSR markers on A03 suggested the A03 resistance locus, *BraA.CR.a* was one of these two resistance loci. To test the newly developed A08 SSR markers, all plants from these five BC_1_ genotypes were analyzed and the data of genotypes and phenotypes showed that the A08 resistance locus, *BraA.CR.b* was the second gene conferring resistance.

### Novel Clubroot Resistance Gene Mapping in ECD3

All seven ECD3 derived BC_1_ families confirmed segregation of A08 resistance locus, *BraA.CR.b*, that suggests locus *BraA.CR.b* is in homozygous state in ECD3. However, previously mapped A03 locus, *BraA.CR.a* did not show co-segregation with clubroot resistance phenotype in ECD3 derived BC_1_ families. Therefore a new clubroot resistance locus was mapped through candidate gene mapping approach. Clubroot resistant BC_1_ individual plants that did not carry the above mentioned clubroot resistance loci were selected to form a resistant plant pool and a susceptible plant pool. Using these two DNA pools, bulk segregant analyses were performed in the same way as described previously. Over 100 SRAP primer pairs were used for bulk segregant analysis with pools containing eight resistant and eight susceptible individual plants, respectively. Two SRAP markers co-segregated with the clubroot resistance phenotype were found to be located on the chromosome A03 on ultra-dense genetic map of *B. rapa* (Li et al., 2011). SRAP markers linked to the third resistance locus were sequenced and the genomic sequence flanking these SRAP markers were identified (Figure 3). A 4 Mb genomic region flanked by these two SRAP markers on chromosome A03 was extracted using BEDtools with Linux commands and blasted against the Arabidopsis genome using blast2go software. At the same time, we also examined annotation information of the *B. rapa* genome database (see text footnote 1) for the 4 Mb genomic region that contains a total 757 genes. The cloned A03 and A08 clubroot resistance genes all belong to the *TIR-NBS-LRR* gene families. Interestingly, the four loci that contained multiple copies of *TIR-NBS-LRR* genes were identified in the 4 Mb regions. All four loci were distinct from the clubroot resistance locus earlier mapped in Chinese cabbage as well as in the BC_1_ populations developed from ECD1, ECD2, and ECD4.

To confirm the third resistance locus in the target region, 50 new SSR markers that covered approximately 18 Mb region of the chromosome A03 including major clubroot resistance genes were developed and used to analyze the ECD3-derived BC_1_ families to investigate linkage with the third clubroot resistance locus. Among these new SSR markers, bulk segregant analyses showed that three SSR markers co-segregated with the clubroot resistant phenotype and remaining markers either did not show polymorphism between resistant and susceptible alleles or did not produce PCR products. These 3 SSR markers, SS15FR1, SS15FR3, and SS15FR5 were screened on all seven ECD3 derived BC_1_ families (Table 6). Three SSR markers co-segregated with the yellow sarson alleles were homozygous in all susceptible plants while the EDC3 alleles of these markers on A03 showed heterozygous with a few individuals missed in ECD3 derived BC_1_ families. It indicated that the A03 resistance locus segregated in the ECD3-derived BC_1_ families (Table 4). These three SSR markers covered the genomic region of chromosome A03 where the novel clubroot resistance locus with three copies of *TIR-NBS-LRR* genes was located. The newly mapped clubroot resistance locus on chromosome A03 was named *BraA.CR.c.* This clubroot resistance locus co-segregated in the BC_1_ families derived from the ECD3 that segregated with a 3:1 ratio (Table 4). The newly mapped A03 resistance locus *BraA.CR.c* was heterozygous while the earlier mapped A08 resistance locus *BraA.CR.b* was homozygous in ECD3 (Table 5).

**Table 4:** Segregation of SSR linked to the new clubroot resistance locus on A03 (located at the 15 Mb physical position of *Brassica rapa* genome) in the BC_1_ populations derived from crosses between clubroot resistant ECD3 accessions and the susceptible yellow sarson in *Brassica rapa*.

Population	Phenotype	Genotype	CR gene markers on A03		
			
			SS15FR1	SS15FR3	SS15FR5
BC1-ECD3	Resistant	Rr	254	255	253
	Susceptible	rr	481	475	475


**Table 5:** Composition of clubroot resistance genes in turnip accessions of the European clubroot differential (ECD) set.

Accessions	Resistance loci		
	
	*BraA.CR.a* (A03)^†^	*BraA.CR.b* (A08)	*BraA.CR.c* (A03)^††^
ECD1	Homozygous	Heterozygous	No
ECD2	Heterozygous	Homozygous	No
ECD3	No	Homozygous	Heterozygous
ECD4	Homozygous	Homozygous	No


*^†^Clubroot resistance R gene BraA.CR.a previously mapped in Chinese cabbage also segregating in some of the ECD accessions (Gao et al., 2014). ^††^Novel clubroot resistance R gene, BraA.CR.c mapped in ECD3.*

**Table 6:** Sequence information of primers used in clubroot gene mapping.

Primer names	Sequence (5′-3′)
S33R33	CACGACGTTGTAAAACGACACTCTCAGCAGGTCACACA
RS33R33	TGCATATCGAGGACATTTG
S30R30	CACGACGTTGTAAAACGACAAAGCAAAGCCATCCTTTA
RS30R30	CGCAAAGTACGAGAATCAA
S27R27	CACGACGTTGTAAAACGACGTTTCCAACTAATCGCATTT
RS27R27	CTGTTTGCCACTAAACCAA
S23R23	CACGACGTTGTAAAACGACTTTCGGAATCCATGAAAGT
RS23R23	CCGCTACACCTCTCACTCT
S18R18	CACGACGTTGTAAAACGACGCTAGCCAACATTTGAACA
RS18R18	ATTCGATCAAACCACACAA
S17R17	CACGACGTTGTAAAACGACCATCCACTTTGGACTGTGA
RS17R17	TCAGAGAACTCAGCTCGTG
S14R14	CACGACGTTGTAAAACGACTTAAATCCGGACGTGAAAT
RS14R14	AAGAGGAAGAAGCTCCTGA
S11R11	CACGACGTTGTAAAACGACACAGAAAGTTGCAAATGACA
RS11R11	TTCACGGGACAGTACAAAA
S08R08	CACGACGTTGTAAAACGACTCCTCAGAGGCTTCTTTTC
RS08R08	TCAATCATTTGTTGCGTTT
S07R07	CACGACGTTGTAAAACGACCCACCCCTACAAACCATAA
RS07R07	TAAACATTGCATGGACCAA
S06R06	CACGACGTTGTAAAACGACCCGAAGTAAACGAGGAGAG
RS06R06	AGATCCGGGTAGAGTCTCA
FSASS45b	CACGACGTTGTAAAACGACGCTATTAAAGTTAGGAATGCT
RSASS45b	GCGCTTAATGAATACTCTCT
FSASS79b	CACGACGTTGTAAAACGACCTTCATAGAGCTAAGCATGT
RSASS79b	ATGTACTGAACCAAGATCCT
FSASS72b	CACGACGTTGTAAAACGACCACAAATTCAATGCAACGCT
RSASS72b	AACAAGTCAATAGTAGT
FSASS45c	CACGACGTTGTAAAACGACTCAACAATCTATGATCCATCT
RSASS45c	TGTAATCTTTAGGTACATACT
SS15FR1	CACGACGTTGTAAAACGACGTATTCGGACATGGGACAT
RSS15FR1	CGAAACCATCTCCTTCTTC
SS15FR3	CACGACGTTGTAAAACGACATCACCACCTTCATCTTCC
RSS15FR3	TCAGTGTGGGGTAAACAGA
SS15FR5	CACGACGTTGTAAAACGACAAGCGTAGTTACGGGAGAG
RSS15FR5	TGGTCGTTACAGTGCATTT
Labeled M13	CACGACGTTGTAAAACGAC


## Discussion

The ECD set is commonly used to investigate the change of clubroot pathogen isolates in the research community of *Brassica* species. The ECD set contains high levels of clubroot resistance known as the progenitors of clubroot resistant Chinese cabbage cultivars and was recently used in the development of oilseed rape cultivars. Since all the ECD accessions are open-pollinated genotypes, they belong to a mixture of individual plants with different clubroot resistance loci. Moreover, accurate phenotyping of clubroot resistance is another challenge. Without accurate phenotyping, only QTL mapping was adequate to analyze the resistance in ECD accessions (Chen et al., 2013). In the current study, using an indoor phenotyping method under controlled environmental conditions with Canadian field isolates, we have successfully mapped three independent clubroot resistance loci on three chromosome regions in all four turnip accessions in the ECD set. Two independent resistance loci on chromosome A03 and one on chromosome A08 were identified in the ECD turnips and also the homozygosity of these three resistance loci in the ECD turnips were analyzed by using multiple BC_1_ families from each of the ECD accessions (Table 5). At the same time, we have developed near iso-genic lines (NILs) containing single clubroot resistance locus in the same genetic background for each mapped locus (data not shown).

**FIGURE 4:**
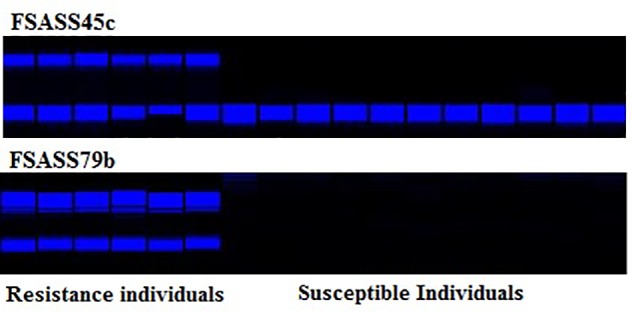
SSR molecular markers, FSASS45c and FSASS79b linked with *BraA.CR.a* on A03 with their co-segregation with clubroot resistance phenotype in BC_1_ populations of *Brassica rapa*.

Since one clubroot resistance gene was fined mapped on chromosome A03 in five Chinese cabbage hybrid cultivars of *B. rapa* (Gao et al., 2014), molecular markers for this locus were available to analyze the clubroot resistance loci in the ECD turnips. According to segregation data of genotypes and phenotypes, this locus *BraA.CR.a* co-segregated with the resistance in the BC_1_ families derived from ECD1, ECD2, and ECD4 (Figure 4). To deal with new clubroot resistance loci, SRAP markers were used to identify the chromosome regions anchoring two newly mapped resistance loci in the ECD set. A candidate gene mapping approach allowed us to pinpoint the genomic regions containing clubroot resistance loci. Candidate clubroot resistance gene on chromosome A08 was located in a 2 Mb chromosome region where about 275 genes were annotated on the *B. rapa* genome database (see text footnote 1). Among these 275 genes, three loci encode putative TIR-NBS-LRR proteins, a typical class of disease resistance proteins. However, two of these three loci were truncated and we were unable to deduce the amino acid sequence for functional proteins based on sequence information of BAC clones. Interestingly, *BraA.CR.b* was identified as a candidate locus involved in clubroot disease resistance and linked SSR markers developed in the ECD set in this study. Twelve SSR markers were polymorphic and closely linked to this gene on chromosome A08, among them, seven were co-dominant and five were dominant (Table 3). In a previous study, Suwabe et al. (2003) mapped two loci, *Crr1* and *Crr2* in parental genotypes “G004” and “Hakusai Chukanbohon Nou 7” of *B. rapa* and revealed complementary action of these loci for clubroot resistance. Hatakeyama et al. (2013) cloned and functionally characterized the *Crr1a* locus in G004 and suggested that this single locus conferred clubroot resistance in *B. rapa* against isolate Ano-01. The clubroot resistance locus mapped in this study may be the same locus as mapped by Hatakeyama et al. (2013). However, gene sequence characterization with function and existence of allelic variations along with their interactions with different isolates of *P. brassicae* need to be conducted to confirm this.

Furthermore, third clubroot resistance locus, *BraA.CR.b* was mapped in the two genes segregating BC_1_ families derived from ECD3 in this study. Although, previous studies reported QTL mapping for clubroot disease in *Brassica* crops (Matsumoto et al., 1998; Piao et al., 2004; Sakamoto et al., 2008; Ueno et al., 2009; Rahman et al., 2011; Kato et al., 2012; Hatakeyama et al., 2013). In contrast, Piao et al. (2004) reported *CRb* likely derived from ECD01 based on survey study. In this study, we identified three clubroot resistance loci on two different chromosomes, which showed qualitative clubroot resistance mechanism against Canadian filed isolate of *P. brassicae*. This suggests that qualitative resistance alone or together with quantitative resistance regulates clubroot disease reactions in *Brassica* species (Piao et al., 2009). Characterization of the clubroot resistance genes, *Crr1* and *CRa* on chromosomes, A08 and A03, respectively, revealed sequence encoding a TIR-NBS-LRR protein, which is usually a product of plant disease resistance R genes (Ueno et al., 2012; Hatakeyama et al., 2013).

The clubroot resistance locus, *BraA.CR.a* was linked with the SSR markers that were previously developed in Chinese cabbage (Gao et al., 2014). Previously, locus *CRb* is believed to be derived from ECD1 (Piao et al., 2004), which might be similar to the locus, *BraA.CR.a* mapped in the current study as well as in Chinese cabbage. Since this locus on chromosome A03 possesses three or six TIR-NBS-LRR genes in the *B. rapa* genome (Hatakeyama et al., 2017), it is difficult to conclude at this point whether it is the same gene copy in all three ECD resistance sources or different functional copies or allelic variations in the same copy. Further investigation is necessary to functionally characterize each copy from different resistance sources. Additional two clubroot resistance loci, *BraA.CR.b* on A08 and *BraA.CR.c* on A03 were mapped using candidate gene mapping approach with ultra-dense genetic map with known SRAP marker sequence data. SRAP markers linked to the clubroot resistance loci were anchored on our ultra-dense genetic map and new polymorphic SSR markers were developed on both chromosomes. Clubroot resistance locus, *BraA.CR.b* on A08 could be similar to previously map locus *Crr1* on A08 (Hatakeyama et al., 2013). However, it is difficult to conclude without knowing sequence information of each genetic locus and its allelic variations among ECDs in this study and their comparison with previously map gene *Crr1a*. Interestingly, location of third clubroot resistance locus, *BraA.CR.c* on A03 was completely different from the previously mapped locus, *BraA.CR.a* on A03 in ECD1, ECD2, and ECD4 in this study and Chinese cabbage described by Gao et al. (2014). In a previous study, origin and inheritance of *CRa* locus was reported from fodder turnip line ECD2 (Matsumoto et al., 2005), which may be similar to locus *BraA.CR.a* on A03 identified in ECD1, ECD2, and ECD4 in this study, and Chinese hybrid previous study by Gao et al. (2014).

## Author Contributions

AH and FG performed the research work of population development and molecular marker analysis. JL and CW tested clubroot resistance. GL and PM designed the experiment. AH and GL wrote the manuscript. RD revised the manuscript. All authors read and approved the final manuscript.

## Conflict of Interest Statement

The authors declare that the research was conducted in the absence of any commercial or financial relationships that could be construed as a potential conflict of interest.

## Footnotes

1 http://brassicadb.org/brad/
